# The presence of the pilus locus is a clonal property among pneumococcal invasive isolates

**DOI:** 10.1186/1471-2180-8-41

**Published:** 2008-02-28

**Authors:** Sandra I Aguiar, Isa Serrano, Francisco R Pinto, José Melo-Cristino, Mario Ramirez

**Affiliations:** 1 Instituto de Microbiologia, Instituto de Medicina Molecular, Faculdade de Medicina Universidade de Lisboa, Lisboa, Portugal

## Abstract

**Background:**

Pili were recently recognized in *Streptococcus pneumoniae *and implicated in the virulence of this bacterium, which led to the proposal of using these antigens in a future pneumococcal vaccine. However, pili were found to be encoded by the *rlrA *islet that was not universally distributed in the species. We examined the distribution of the pilus islet, using the presence of the *rlrA *gene as a marker for the locus, among a collection of invasive isolates recovered in Portugal and analyzed its association with capsular serotypes, clusters defined by the pulsed-field gel electrophoretic profiles (PFGE) and multilocus sequence types.

**Results:**

Only a minority of the isolates were positive for the presence of the *rlrA *gene (27%). There was a high correspondence between the serotype and the presence or absence of *rlrA *(Wallace coefficient, W = 0.778). In particular, there was an association between the presence of *rlrA *and the vaccine serotypes 4, 6B, 9V and 14 whereas the gene was significantly absent from other serotypes, namely 1, 7F, 8, 12B and 23F, a group that included a vaccine serotype (23F) and serotype 1 associated with enhanced invasiveness. Even within serotypes, there was variation in the presence of the pilus islet between PFGE clones and a higher Wallace coefficient (W = 0.939) indicates that carriage of the islet is a clonal property of pneumococci. Analysis of *rlrA *negative isolates revealed heterogeneity in the genomic region downstream of the *rfl *gene, the region where the islet is found in other isolates, compatible with recent loss of the islet in some lineages.

**Conclusion:**

The pilus islet is present in a minority of pneumococcal isolates recovered from human invasive infections and is therefore not an essential virulence factor in these infections. Carriage of the pilus islet is a clonal property of pneumococci that may vary between isolates expressing the same serotype and loss and acquisition of the islet may be ongoing.

## Background

Non-flagellar polymeric cell-surface organelles designated as pili were initially identified in Gram-negative bacteria. In the last decade, pilus-like surface structures have been described in Gram-positive bacteria like *Corynebacterium *spp., *Actinomyces *spp. and several streptococcal species [1], but only recently were pili identified in the major pathogenic species of the genus: *Streptococcus pyogenes*, Lancefield group A [2]; *Streptococcus agalactiae*, Lancefield group B [3] and *Streptococcus pneumoniae *(pneumococcus) [4].

In *S. pneumoniae *pili are encoded by the *rlrA *pathogenicity islet, a 14.2 kb region composed of 7 genes encoding a putative transcriptional regulator (*RlrA*), 3 LPXTG surface proteins with weak homology to microbial surface components recognizing adhesive matrix molecules – MSCRAMMs (RrgA, RrgB and RrgC) and 3 sortases (SrtB, SrtC and SrtD) [4-6]. Pneumococcal pili have the appearance of flexible rods ranging from 0.3 μm to 3 μm that are formed by a transpeptidase reaction involving sortase-mediated covalent cross-linking of the proteins containing the LPXTG-like motif and several lines of evidence suggest a one-to-one sortase-to-surface-protein correspondence for the proteins encoded by the islet [7].

The *rlrA *gene was identified in the original signature tagged mutagenesis study as an essential virulence gene in murine models of infection [5]. Later studies confirmed that the *RlrA *protein acted as a transcription factor recognizing several promoters within the *rlrA *islet and showed it to be essential for wild-type levels of expression of the pili structural genes and associated sortases [6]. The product of the *mgrA *gene, located outside the *rlrA *islet, was shown to act as a transcriptional repressor of the islet genes, including *rlrA*, being responsible for the silencing of the locus in the absence of *RlrA *[8].

The presence of the *rlrA *pathogenicity islet was shown to influence pneumococcal capacity to adhere to human lung epithelial cells [4,8]. Mouse models of pneumococcal pneumonia and bacteremia have also suggested a role of pili in virulence and host inflammatory responses [4,5]. More recently, immunization of mice with pilus structural antigens was shown to induce protection against lethal challenge by piliated strains [9]. Moreover, these studies indicate that vaccination with the pilus subunits offers the same protection as vaccination with heat killed bacteria, raising the possibility of using pilus antigens in a multivalent pneumococcal vaccine [9].

In spite of these favorable results, early genomic studies indicated that the *rlrA *islet was not present in all pneumococcal isolates, suggesting that it could have been acquired by horizontal gene transfer [10] and raising a cautionary note concerning the use of pilus antigens in any future vaccine. A survey for the distribution of the sortase genes identified the *srtA *gene in all tested bacteria, but the sortases associated with the *rlrA *islet were present in only 17% of the isolates tested [11]. A recent study, specifically designed to evaluate the distribution of the *rlrA *islet among pneumococcal isolates associated with colonization and infections in Native Americans, found that only 21% of the isolates were positive for *rrgC*, a gene encoding a pilus structural protein, and that there was an association between the presence of this gene and the serotypes included in the seven-valent conjugate vaccine [12]. However, the same study could not show a difference in the presence of *rrgC *between the isolates recovered from the nasopharynx and those recovered from sterile sites. In contrast, the *mgrA *gene encoding the transcriptional repressor of the *rlrA *islet seems to be universally distributed within *S. pneumoniae *[8].

In view of these findings, a better understanding of the distribution of the pilus islet in the invasive pneumococcal population will help in the evaluation of its proposed role as a virulence factor and as a potential vaccine candidate. In the present study, we determined the prevalence of the *rlrA *gene in a recent collection of invasive isolates from both children and adults with the aim of identifying associations between the presence of the pilus islet and serotype, antimicrobial resistance or clonal types defined by pulsed-field gel electrophoresis (PFGE) and multilocus sequence typing (MLST).

## Results

### Distribution of the *rlrA *islet among invasive pneumococci

Only a small portion (27%) of our invasive isolates was *rlrA *positive and a total of 355 (73%) isolates were found to lack the pilus islet by Southern blot hybridization and PCR amplification of the *rlrA *gene (Additional file 1: Southern hybridization of a representative set of isolates with the *rlrA *gene probe.). A group of 39 isolates representative of the diversity found among the *rlrA *positive isolates (n = 128) was subject to long-range PCR. All isolates yielded fragments larger than 14 kb compatible with the presence of the entire pilus locus (data not shown), confirming that the presence of the *rlrA *gene is a good marker for the presence of the entire pilus islet.

**Figure 1:**
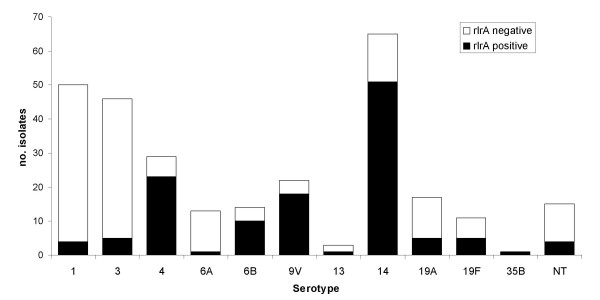
**Frequency of the *rlrA *islet among serotypes in which at least one *rlrA *positive isolate was found**. Black bars indicate the number of isolates positive for the presence of the *rlrA *islet. White bars indicate the number of isolates negative for the presence of the *rlrA *islet.

Overall *rlrA *positive isolates were found in 11 serotypes of the 41 serotypes in our collection (Additional file 2: Characteristics of the clones where the pilus locus was identified.). Isolates expressing serotypes 1, 3, 4, 6A, 6B, 9V, 13, 14, 19A, 19F, 35B were shown to carry the *rlrA *gene as well as non-typable isolates, but 83% of the *rlrA *positive isolates expressed vaccine serotypes (4, 6B, 9V, 14, and 19F). The proportion of isolates carrying the pilus islet among isolates of the same serotype was variable (Figure 1), but overall serotype was a good predictor of the presence or absence of the pilus islet, as indicated by the high Wallace coefficient (W = 0.778).

Although there were differences in the overall proportion of isolates carrying the pilus islet between the isolates recovered from children < 6 yrs. old (37%) and from adults (24%) (p = 0.026), we could not shown differences between the two age groups when we considered the proportion of *rlrA *positive isolates stratified by serotype using the Mantel-Haenszel test (p = 0.200), indicating that the overall disparity is due to a different distribution of serotypes in the two age groups.

In order to identify which serotypes were associated with the presence of the *rlrA *islet, odds ratios were calculated. An OR > 1 implies that the serotype is associated with the presence of *rlrA*, while an OR < 1 indicates that the serotype is significantly depleted of *rlrA *positive isolates. According to this analysis, serotypes 4, 6B, 9V and 14 were highly associated with the presence of the pilus islet (p < 0.01 for all serotypes). On the other hand, the *rlrA *islet was significantly absent from serotypes 1, 7F, 8, 12B and 23F (Table 1).

**Table 1:** Association of the *rlrA *islet with serotypes and PFGE clusters

**Serotype**	**OR (95% CI)^a^**	**p**	**STs in PFGE cluster^b^**	**OR (95% CI)**	**p**
1	0.22 (0.07 – 0.53)	0.0011	[ST306 + ST228]	0.079 (0.01 – 0.59)	0.0007
3	NS^c^		[ST1220 + ST260] + [ST1230 + ST180]	0.06 (0.01 – 0.44)	< 10^-4^
4	12.74 (5.05 – 32.12)	< 10^-4^	ST1221	80.26 (4.72 – 1363.4)	< 10^-4^
			ST247	30 (3.8 – 236.84)	< 10^-4^
6B	7.44 (2.29 – 24.16)	0.0005	ST1224 + ST273	47.33 (2.7 – 830.37)	< 10^-4^
7F	0 (0 – 0.61)	0.0052	ST191	0 (0.0 – 0.70)	0.0089
8	0 (0 – 0.37)	0.0002	ST53	0 (0.0 – 0.57)	0.0057
9V	14.36 (4.76 – 43.33)	< 10^-4^	[ST156 + ST557 + ST644 + ST1225]	16.76 (4.80 – 58.54)	< 10^-4^
12B	0 (0 – 0.70)	0.0089	NS^c^	NS^c^	
14	16.13 (8.50 – 30.63)	< 10^-4^	[ST156 + ST557 + ST790]	212.4 (28.89 – 1561.8)	< 10^-4^
23F	0 (0 – 0.44)	0.0006	ST338 + ST1371	0 (0.0 – 0.57)	0.0057

^a^An OR of > 1 indicates increased proportion of *rlrA *positive isolates whereas OR of < 1 indicates decreased proportion of *rlrA *positive isolates. Only statistically significant results (FDR < 0.05) are presented.

^b^STs in PFGE clusters with a Dice similarity coefficient of > 80%. Brackets indicate STs that belong to the same lineage, as defined by eBURST analysis with the complete *S. pneumoniae *database available at spneumoniae.mlst.net. Only statistically significant results (FDR < 0.05) are presented.

^c^NS – not significant

### Association between the presence of the pilus islet and resistance to antimicrobials

A clear correlation between the presence of *rlrA *and antimicrobial resistance was noted. We found that most isolates carrying the pilus islet were resistant to at least one antimicrobial – 54% (69/128) were non-susceptible to penicillin, 18% (23/128) were resistant to erythromycin and 17% (22/128) were multi-resistant. This association was further confirmed since the OR calculated for resistance to individual antibiotics and the presence of the pilus islet were mostly > 1 and significant (Table 2).

**Table 2:** Association of the *rlrA *islet with antimicrobial resistance

**Antibiotic**	**no. rlrA positive strains/no. resistant isolates (% rlrA positive strains)**	**no. rlrA positive strains/no. susceptible isolates (% rlrA positive strains)**	**OR(95% CI)^a^**	**p**
**Penicillin^b^**	69/113 (61)	59/370 (16)	8.27 (5.17 – 13.22)	< 10^-4^
**Erythromycin**	23/48 (48)	105/435 (24)	2.89 (1.58 – 5.30)	0.0004
**Tetracycline**	16/38 (42)	112/445 (25)	2.16 (1.09 – 4.26)	0.023
**Chloramphenicol**	5/14 (36)	123/469 (26)	NS^c^	
**Co-trimoxazole**	74/106 (70)	54/377 (14)	13.83 (8.35 – 22.91)	< 10^-4^
**MDR^d^**	22/42 (52)	106/441 (44)	3.48 (1.83 – 6.62)	< 10^-4^

^a^An OR of > 1 indicates increased proportion of *rlrA *positive isolates whereas OR of < 1 indicates decreased proportion of *rlrA *positive isolates. Only statistically significant results (FDR < 0.05) are presented.

^b ^Both penicillin intermediate and fully resistant isolates were considered resistant for this analysis

^c^NS – not significant

^d^MDR – multidrug resistance defined as resistance to at least three different antimicrobial classes considering as resistant to penicillin both intermediate as well as fully resistant isolates

### Presence of the pilus islet is a clonal property

Characterization of the genetic lineages of the pneumococcal collection included in the present study had been performed previously [13]. Analysis by genetic lineage of the isolates demonstrated that the presence of the *rlrA *islet was not only associated with serotype but also with PFGE cluster within each serotype, defined as described previously [13] (Additional file 2 – Characteristics of the clones where the pilus locus was identified – and Additional file 3 – Characteristics of the bacterial clones not associated with the pilus locus). The Wallace coefficient relating the PFGE clusters with the presence or absence of *rlrA *(W = 0.939) was higher than for serotype (W = 0.778) indicating that the pathogenicity islet is clonally distributed. The value of the Wallace coefficient indicates that only one out of 20 pairs of isolates grouped in the same PFGE cluster will differ in the presence of the *rlrA *islet in their genome.

To verify if there were PFGE clusters associated with the presence of *rlrA*, odds ratios were determined and the significant values obtained in this analysis are indicated in Table 1. In some serotypes identified as associated with the presence of the pilus islet, all of the major PFGE clusters showed also a positive association with the presence of *rlrA *(serotype 4, table 1 and Additional file 2: Characteristics of the clones where the pilus locus was identified.), but in others only some of the PFGE clusters were associated with *rlrA *(serotype 14, table 1 and Additional file 2: Characteristics of the clones where the pilus locus was identified). The fact that the presence of the *rlrA *islet is a clonal property is further illustrated by isolates of serotype 3, that is not itself significantly associated with the presence nor with the absence of the *rlrA *islet, but a clone expressing serotype 3 was found to be significantly depleted of the pilus islet (table 1). Note that the OR values are more extreme when compared with the values calculated for serotype alone, further supporting the notion that the presence of the pilus islet is a clonal property.

Prior work from our group showed that there is a close correspondence between PFGE cluster and ST [13]. The relationships between sequence types have been explored by various methodologies, including eBURST [14] that was specially developed to analyze MLST data and is frequently used to define clonal complexes. An eBURST analysis revealed that all isolates carrying the pilus islet expressing serotypes 9V and 14, which represented 54% (69/128) of the total isolates positive for *rlrA*, were clonally related and represented clone Spain^9V^-3 (ST156) [15] (Additional file 2: Characteristics of the clones where the pilus locus was identified.). Another association of the presence of the pilus islet and PMEN clones was observed in serotype 6B where all seven isolates belonging to a PFGE cluster represented by ST1224 and ST273 and a single isolate in a different PFGE cluster, but also exhibiting ST273, were all positive for the presence of *rlrA *and are related to clone Greece^6B^-22 [16] (Additional file 2: Characteristics of the clones where the pilus locus was identified.). On the other hand, among serotype 14 isolates related to England^14^-9 (PFGE cluster represented by ST15 and ST409), and serotype 6B isolates related to Poland^6B^-20 (PFGE cluster characterized by ST887), no isolates carrying the pilus islet could be found.

### Characterization of *rlrA *negative isolates

**Figure 2:**
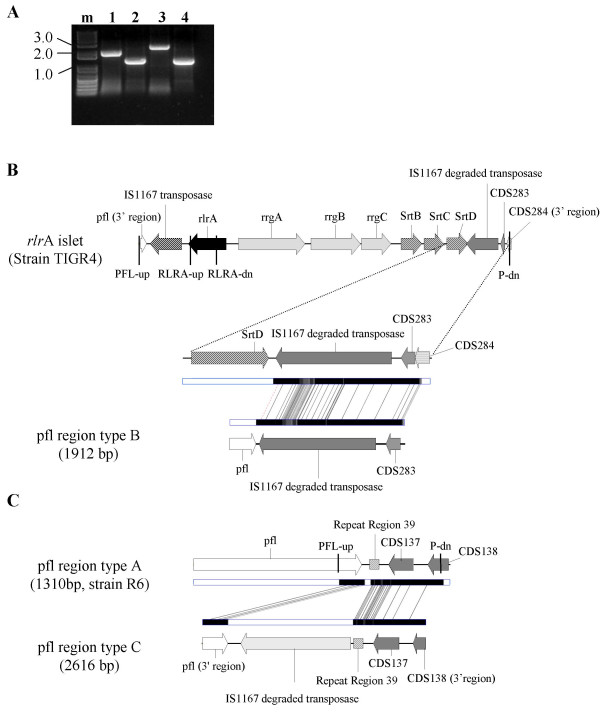
**The genetic structure of the region downstream of the *pfl *gene where the *rlrA *islet is found among piliated isolates**. **Panel A. **Agarose gel of the PCR products resulting from the amplification of the region downstream of the *pfl *gene using primers PFL-up and P-dn. m – DNA ladder (1 kb plus, Invitrogen, Carlsbab, CA). Lane 1 – isolate presenting a region type C. Lane 2 – isolate presenting a region type A. Lane 3 – isolate presenting a region type B. Lane 4 – strain R6. Sizes of the fragments in kilobases are indicated in the left. **Panel B. **Structure of the region found downstream of the *pfl *gene in four isolates and designated region type B compared to the *rlrA *islet and flanking regions of strain TIGR4. Genes are indicated by arrows and primers by vertical lines. Genes encoding proteins with similar functions are represented by the same pattern. The fragments with high DNA similarity between the regions compared are represented by black and grey boxes connected by lines (analysis was performed using the GATA software). **Panel C. **Structure of the region found downstream of the *pfl *gene in 68 isolates and designated region type C compared to the corresponding region of strain R6 (region type A).

The majority of the isolates were negative for the presence of the *rlrA *gene (n = 355). To explore the possibility that the *rlrA *negative isolates still retained parts of the pathogenicity islet, a PCR was performed using a set of primers that flanked the whole islet (Figure 2B and 2C, primers PFL-up and P-dn). All isolates yielded a PCR product and the majority (283 isolates, 80%) originated a product with the same size as strain R6 (Figure 2A, lanes 2 and 4 and figure 2C). The remaining 72 (20%) isolates revealed larger amplification products: 68 presented a product designated type C (figure 2A, lane 3 and figure 2C), while the remaining 4 presented a product designated type B (Figure 2A, lane 1 and figure 2B). In order to determine the genetic differences responsible for the heterogeneity in size of the PCR products, a representative isolate of types B and C was sequenced. The genetic makeup of the various regions is illustrated in figures 2B and 2C.

Both the type B and C regions, contain DNA stretches similar to those found in the 3' region of the *rlrA *locus of strain TIGR4. These common DNA elements were found to be similar to the region found upstream of the *rlrA *locus of TIGR4 encoding the IS1167 transposase however, due to mutations and indels, these are no longer capable of producing a full-length functional protein. BLAST searches demonstrated a high number of similarly degraded copies of the IS1167 transposase distributed throughout the available complete genomes of strains of Streptococcus pneumoniae (TIGR4, R6 and D39). There is a high identity between the IS1167 degraded transposase sequence found downstream of the *rlrA *locus in TIGR4 and the one found in the type B region (97% identity in the 1277 nt aligned, with only two single nucleotide gaps). The similarity extends to other coding and non-coding regions found between srtD and spr0470 of TIGR4 (Figure 2B). This observation is compatible with the hypothesis that region type B was generated by the recent loss of the *rlrA *islet. Further supporting this notion is the observation that this region was found exclusively among the few isolates not carrying the pilus islet among large PFGE clusters of *rlrA *positive isolates (Additional file 2: Characteristics of the clones where the pilus locus was identified.).

The DNA sequence corresponding to the degraded copy of the IS1167 transposase in region type C differed more extensively from the two discussed above, namely by a 50 nt insertion and a 16 nt deletion. A comparison of the type C region with the corresponding sequence of strain R6 revealed extensive similarity, including the presence of ORF CDS137 and a downstream repeat region found in strain R6 but absent from the pilus locus in strain TIGR4 (Figure 2C). The type C region is therefore closely related to the one found in strain R6 with the exception of the presence of a degraded copy of the IS1167 transposase, suggesting that the region downstream of the pfl gene may constitute a hotspot for recombination events. The type C region was found associated with specific PFGE clusters expressing a limited number of serotypes (Additional file 2 – Characteristics of the clones where the pilus locus was identified – and Additional file 3 – Characteristics of the bacterial clones not associated with the pilus locus.), but this was not an exclusive association since isolates presenting regions type A and C were frequently grouped in the same PFGE cluster. For instance, the largest PFGE cluster in serotype 3 contains almost equal numbers of isolates presenting each of the regions.

## Discussion

The long-term efficacy of the seven-valent conjugate vaccine is being challenged by the emergence of infections caused by replacement serotypes not targeted by the vaccine. The difficulty in producing higher valency vaccines and the realization that any polysaccharide based vaccine may be ultimately limited in its efficacy, due to the large number of pneumococcal capsular types, prompted the search for other vaccine targets. Recently, the three structural proteins constituting pneumococcal pili were shown to protect mice against invasive infections and were proposed as potential vaccine candidates [9].

The data presented here argues that the efficacy of such a vaccine would be limited by the small proportion of isolates carrying the pilus islet (27%) and therefore potentially expressing pili, among those causing invasive infections in humans. The strong association of the *rlrA *islet with four of the seven serotypes included in the conjugate vaccine would further limit the advantages of a pilus component vaccine over the one currently available. Recently, Basset et al. explored the distribution of the *rlrA *islet, using *rrgC *as a marker for the presence of the pilus locus [12]. The population analyzed by Basset et al. differed markedly from the one analyzed here by including a large fraction of isolates associated with colonization, a different distribution of serotypes and, most importantly, the genetic makeup of the pneumococcal population was strikingly different with only 15 common STs out of the 158 STs identified overall in the two collections. In spite of these differences, a surprisingly similar proportion of isolates carrying the pilus islet was found among invasive isolates by Basset et al. (24%, n = 123). Taken together the results of the two studies suggest that the pilus does not represent an essential virulence factor for invasive disease in humans, in contrast to previous indications from mouse models [4,5]. This suggestion receives further support from the observation that the same fraction of isolates associated with asymptomatic carriage were positive for the pilus islet [12].

The collection characterized here was previously analyzed for the phase variant expressed by the isolates [17], another phenotype implicated in enhanced invasiveness. A proportion of opaque variants similar to the one positive for the *rlrA *gene was found (26%), prompting us to evaluate the association between opacity and the pilus islet. However, no association could be shown between the opacity phenotype of a given isolate and carriage of the pilus islet. In fact, two serotypes found to be associated with predominantly opaque variants, serotype 1 and 4 [17], were found to be significantly depleted of isolates positive for *rlrA *and strongly associated with the presence of the islet, respectively.

A prior study emphasized the higher prevalence of the pilus islet among the serotypes included in the currently available conjugate vaccine versus all other serotypes [12]. Here we expand these findings by identifying which serotypes are particularly enriched or depleted in the presence of the pilus locus. Specifically, among the seven serotypes included in the conjugate vaccine, although most serotypes are indeed associated with the presence of the pilus locus (4, 6B, 9V and 14), serotype 23F was significantly depleted of the presence of this locus, indicating that an association with the presence of the pilus locus is not a property of all vaccine serotypes, while serotypes 18C and 19F did not reach statistical significance. Interestingly, while vaccine-serotype 14 was associated with the presence of the pilus islet in the present study, the islet was found in less than 10% of the isolates expressing this serotype among a collection of North American isolates [12]. This discrepancy is possibly due to differences in the genetic structure of this serotype in the two collections, as discussed below, and highlights the pitfalls of establishing an association between the presence of the pilus locus and serotype without consideration for the clonal structure of the bacterial population expressing a particular serotype. It is also noteworthy that serotype 1, previously identified as having enhanced invasive disease potential [18], was significantly depleted of isolates carrying the pilus islet (table 1) in contrast to the proposed role of pili as virulence potentiators.

We could also show an association between the presence of the pilus islet and resistance to several antimicrobials, including penicillin (table 2). Such an association was expected since the serotypes where the pilus islet was found also concentrate the majority of resistant isolates. Recently, pili were suggested to play an important role in the dissemination of penicillin non-susceptible isolates in Sweden [19]. The clonal lineage implicated in Sweden was represented by ST156 that was also found to be significantly associated with the presence of the pilus islet in our collection, independently of the serotype expressed by the isolates (serotype 9V or 14, table 1).

An analysis of the genetic backgrounds of the isolates showed that the best predictor of the presence of the pilus islet was the PFGE cluster, independently of the serotype, indicating that this is a clonal property. Particularly interesting, is the fact that different lineages expressing the same serotype can be associated with the presence or absence of the locus, which may confound an analysis of association of the pilus islet with serotype only disregarding the clonal structure of the population. For instance, among the isolates expressing serotype 14, the dominant lineage characterized by ST156 and related STs was associated with the presence of the pilus locus in a serotype independent analysis (table 1). However, in the second most prevalent lineage expressing serotype 14, characterized by ST15 and related STs, the locus is conspicuously absent (Additional file 2: Characteristics of the clones where the pilus locus was identified.). A different clonal structure could thus explain the low prevalence (< 10%) of the pilus locus in serotype 14 in the report of Basset et al. [12] that is in sharp contrast with the high prevalence of the locus in this serotype found in our collection (78%, Additional file 2: Characteristics of the clones where the pilus locus was identified.). Importantly, we also show that some PFGE clusters are significantly depleted in isolates carrying the pilus locus and, since these were the dominant clones among their serotypes, a significant depletion at the serotype level was also evident in most cases (table 1). The only exception was serotype 3 where, in spite of the presence of a dominant clone significantly depleted of the pilus locus, the presence of a minor cluster represented by ST458 constituted exclusively by isolates positive for the pilus islet prevented the association at the serotype level to reach significance. The existence of clones expressing the same serotype associated or depleted in the pilus islet raises the possibility that in other geographic areas with a different clonal structure, the association between particular serotypes and the presence or absence of the pilus locus may be different from the one reported here.

A prior study had suggested a strong association between the PMEN clones and the presence of the pilus islet [12]. In the present collection, the Spain^9V^-3 clone was found to be strongly associated with the presence of the pilus islet similarly to what was found previously [12,19], but other PMEN clones found in our collection were devoid of isolates positive for *rlrA *such as England^14^-9 and Poland^6B^-20, so that carriage of the pilus locus is not a universal property of widely disseminated lineages.

Basset et al. discuss the limitations of using the detection of *rrgC *by PCR as a marker for the presence of the pilus islet [12]. One of the concerns is that this may have been a too broad criterion in that strains that carry the *rrgC *gene may lack other genes essential for pilus expression. Although our study suffers from the same limitation, the long-range PCR performed on a subset of the isolates positive for the *rlrA *gene yielded products whose sizes were compatible with the presence of the entire locus, in agreement with previous studies that indicated that the *rlrA *islet is either present or absent in its entirety [12,19]. However, the primary concern of Basset et al. was that theirs could have been a too stringent criterion, since strains not containing the *rrgC *gene or negative by PCR due to variability in this structural gene may still express pili. Our choice of using the presence of *rlrA *to define piliated isolates was designed to avoid these problems. RlrA is a regulatory protein and it is therefore expected to be more conserved than a structural protein and the presence of this protein was shown to be essential for wild-type levels of expression of the pilus structural genes and associated sortases [6], indicating that isolates lacking this gene would also be impaired in pilus expression. Moreover, the PCR performed using primers targeting conserved DNA regions flanking the *rlrA *locus conclusively showed that all strains lacking the *rlrA *gene also lacked all the sortases and structural genes necessary for pilus biosynthesis. Therefore, the 27% of invasive isolates containing the *rlrA *gene constitute the fraction genetically equipped to express pili, although only the availability of specific antibodies targeting the pilus could determine if this genetic potential was fully realized.

The study of the *rlrA *negative isolates revealed different genetic organizations of the region where the pilus islet is usually found, suggesting that this region may be a hotspot for DNA insertions and deletions. Transposons and IS elements have been shown to be a frequent cause of duplications and deletions in bacterial chromosomes through homologous recombination [20]. Significantly, the sequence of the IS1167 transposase retained in the 3' region of the pilus locus is severely altered when comparing to the one in the 5' region (fig. 2) which may hinder homologous recombination and stabilize the presence of the pilus locus. Notwithstanding, the distribution and genetic arrangement of region type B, with the presence of CDS283 that was exclusively associated with the pilus locus, suggests that it may have resulted from the recent loss of the pilus islet in some lineages, possibly through intra-chromosomal homologous recombination between the conserved 3' regions of the flanking IS1167 transposase encoding segments or through an error in excision mediated by a functional IS1167 transposase, such as the one putatively encoded in the 5' region of the locus. A recombination event would be expected to retain most of the 3' copy of the IS1167 transposase and the downstream region as was indeed verified (Fig. 2). The DNA sequence of the type C region, that differs from the more common type A region found in strain R6 by the presence of a degraded IS1167 transposase encoding segment, offers limited clues as to its origin. However, the presence of an IS1167 sequence could further promote the acquisition of the pilus islet by providing a region of sequence similarity that could facilitate homologous recombination.

Carriage of the pilus islet was shown to be a clonal property and its association with a widely disseminated pneumococcal clone (Spain^9V^-3), may indicate that pili could facilitate dissemination or colonization of the host, as suggested recently [19]. The pneumococcal population included in our study was obtained from a period prior to the introduction of the heptavalent vaccine [13,21]. Since the majority of the isolates positive for the pilus locus expressed vaccine serotypes, a decrease of piliated strains due to vaccine introduction, which was already observed in the USA [12], might be expected. However, some serotypes that are emerging due to vaccine pressure, such as 19A [22,23], include clones that carried the *rlrA *islet and the diversity of the region associated with the pilus locus suggests that loss and acquisition of the islet may be ongoing. Since serotype prevalence is changing as a consequence of vaccine introduction [22,23] the acquisition of the pilus locus by the emerging clones or the increase in incidence of piliated clones may shed light on the role of this surface structure in human infections.

## Conclusion

The low prevalence of the *rlrA *islet in our collection of invasive isolates indicates that the pilus does not represent an essential virulence factor in human infections and that its potential use in a vaccine would offer limited benefits. Carriage of the pilus islet was shown to be a clonal property that may vary between isolates expressing the same serotype. The variable nature of the region downstream of the *pfl *gene in isolates lacking the pilus islet suggests that acquisition and loss of the islet may be ongoing.

## Methods

### Bacterial isolates

A collection of 465 invasive pneumococcal isolates recovered during the years of 1999 to 2002 in Portugal were previously described [21]. Additionally, 18 invasive isolates, recovered during the same period, were added to our collection and further characterized (total n = 483). Briefly, 87 isolates were recovered from children < 6 yrs. and 381 from adults (≥ 18 yrs). Overall, serotypes 14, 1, 3, 4, 8, 9V, 23F, 7F, 19A and 12B were the most prevalent by decreasing rank order. The isolates were characterized by a combination of macrorestriction profiling using the SmaI endonuclease and pulsed-field gel electrophoresis (PFGE) and multilocus sequence typing (MLST) [13]. The isolates were analyzed separately by serotype and PFGE-based clusters were defined as isolates with ≥ 80% relatedness on an UPGMA dendrogram constructed using the Dice coefficient. MLST analysis was performed for at least one isolate in each major PFGE cluster (n = 127) and revealed 66 different sequence types (ST) corresponding to 47 different lineages by eBURST analysis [13]. Importantly, a comparison of the clones found in this collection with those found in the United States by Gertz et al. [24] showed that only 11 lineages, as defined by MLST, were common out of a total in excess of 150 STs identified in the two studies. Similarly, a comparison with the study of Basset et al. [12] revealed that only 15 out of the 158 STs identified overall in the two studies were common.

The reference strains TIGR4 [10] and R6 [25] were used as positive and negative controls for the presence of the pilus islet.

### Detection of the *rlrA *islet

Identification of isolates carrying the *rlrA *islet was performed by southern blot hybridization. Genomic DNA from *S. pneumoniae *was prepared in agarose plugs as described previously, digested with SmaI and separated by PFGE in a CHEF-DR II apparatus (Bio-Rad Laboratories, USA) [13]. Following electrophoresis, the DNA was transferred to nylon membranes (Hybond-N^+^, Amersham Biosciences, Bucks, UK) with a VacuGene system (Pharmacia Biotech AB, Uppsala, Sweden) and subjected to Southern hybridization under stringent conditions. The probe was constructed using primers *RLRA*-up (5'-TCT GAT AGA TGA GAC GCT GTT G-3') and *RLRA*-dn (5'-CTC CGC TTC TTT CTA CTA CAA G-3'), which allowed the amplification of an 1177 bp internal fragment of the *rlrA *gene using DNA from strain TIGR4 as template (Figure 2B). Labeling and hybridization were performed using ECL direct nucleic acid labeling and detection system (GE Healthcare, Buckinghamshire, UK), according to the instructions of the manufacturer. The molecular size of the hybridization signals and the corresponding SmaI fragments were determined.

For some isolates hybridization yielded ambiguous results, possibly due to problems during blotting of the DNA onto the nylon membranes or degradation of the membranes during storage. In these cases, the *rlrA *gene was detected by PCR, using *RLRA*-up and *RLRA*-dn primers. Template DNA was prepared by diluting 9 μl of an overnight culture in 441 μl of water and boiling of this mixture for 2 min. The PCR reactions were performed in a 50 μl volume containing 20 μl of template solution, 1× reaction buffer (Biotools, Madrid, Spain), 1.25U Tth DNA polymerase (Biotools, Madrid, Spain), 10 mM dNTPs (Fermentas, Vilnius, Lithuania) and 20 pmol of each of the primers. The PCR program consisted of 30 cycles of 95°C for 30 s, 55°C for 30 s, and 72°C for 30 s, followed by 10 min incubation at 72°C.

### Characterization of the chromosomal region where the *rlrA *islet is usually found in non-piliated strains

In order to confirm that the *rlrA *negative strains did not carry the pilus pathogenicity islet or parts of it, a set of primers that flanked the *rlrA *pathogenicity islet were developed – PFL-up (5'-ATC TCA TTG ACT ACA CAA GTA TCA CCT C-3') and P-dn (5'-CAA GAG CAT ACT CCA ACT CAT AAA TAT GTG-3') – with the aim of amplifying the entire pilus islet (Figure 2B and 2C). All strains that were negative for the presence of the *rlrA *gene were subject to PCR with primers PFL-up and P-dn, as well as TIGR4 (piliated control), and R6 (non-piliated control). If the pilus islet or parts of it were absent, the PCR product expected using these primers would be similar to that obtained when using R6 DNA as template (1310 bp). The PCR reactions were performed in a 50 μl volume containing 20 μl of template solution, 1× reaction buffer (Biotools, Madrid, Spain), 1.25U Tth DNA polymerase (Biotools, Madrid, Spain), 10 mM dNTPs (Fermentas, Vilnius, Lithuania) and 20 pmol of each of the primers. The PCR program consisted of 30 cycles of 95°C for 30 s, 60°C for 30 s, and 72°C for 3 min, followed by 10 min incubation at 72°C. Whenever no PCR product was obtained using these conditions, and in a subset of the isolates positive for the *rlrA *gene representing the diversity found within the collection, the reaction was performed in conditions allowing the amplification of fragments larger than 10 kb. In this case DNA was extracted using CTAB [26] and the PCR reaction was prepared using the Expand Long Template PCR System (Roche, Mannheim, Germany) according to the manufacturers instructions. The PCR program consisted of incubation at 94°C for 2 min followed by 10 cycles of 94°C for 10 s, 58°C for 30 s, and 68°C for 13 min, and 20 cycles of 94°C for 15 s, 58°C for 30 s, and 68°C for 13 min with an incremental increase of 20 s per cycle, followed by 7 min incubation at 68°C.

Strains representative of the various sizes of PCR products obtained were sequenced by primer walking. The sequences were compared using BLAST against the available sequence databases at the NCBI to identify similar sequences and to each other using the MEGA [27] and GATA [28] software programs.

### Statistical analysis

Wallace coefficients (W) were used to compare partitions. This coefficient indicates the probability that two isolates sharing the same characteristic by a given typing method will also be grouped together by a different typing method [29]. In this study, Wallace coefficients were used to estimate the power of a given typing method in predicting the presence or absence of the pilus locus.

Statistical associations between the presence of the *rlrA *islet and serotype, PFGE cluster or antimicrobial resistance profile were characterized by odds ratios (OR) with 95% Wald confidence intervals (CI) [30]. For null OR, 95%CI were computed through the Fisher method [31]. OR significance or statistical differences between proportions were tested with the chi-square statistic. The resulting p-values were corrected for multiple testing by controlling the False Discovery Rate (FDR) under or equal to 0.05 through the linear procedure of Benjimini and Hochberg [32]. Difference in proportion of *rlrA *islet presence in isolates recovered in different age groups, stratifying for serotype was evaluated with the Mantel-Haenszel test [33]. The p-values indicated in the text were considered significant if lower than 0.05.

Wallace coefficient analysis was done in a web application [34] and the remaining statistical tests were executed in the R statistical language using functions implemented in the epitools package [35].

### Nucleotide sequence accession numbers

The sequences of isolates representing the various sizes of the PCR products obtained in *rlrA *negative isolates were deposited in GenBank with accession numbers EU126839 and EU126840.

## Authors' contributions

SIA and IS carried out molecular studies. FRP performed the statistical analysis. SIA and FRP drafted the manuscript. JMC participated in the design of the study and helped to draft the manuscript. MR conceived the study, and participated in its design and coordination and helped to draft the manuscript. All authors read and approved the final manuscript.

## Supplementary Material

Additional file 1 **Southern hybridization of a representative set of isolates with the *rlrA *gene probe**. m, lambda PFGE ladder marker (New England Biolabs, Beverly, MA). Lane 1, strain R6. Lanes 2–4, 6 and 8, isolates lacking the pilus islet. Lanes 5 and 9–14, isolates positive for the presence of the *rlrA *gene. Lane 7, isolate with a weak hybridization signal where the presence of the islet was confirmed by PCR. In all isolates with a negative hybridization result the absence of the pilus islet was confirmed by PCR (see text). Click here for file

Additional file 2 Characteristics of the clones where the pilus locus was identified. Click here for file

Additional file 3 Characteristics of the bacterial clones not associated with the pilus locus. Click here for file
