# Supplementary material for: The presence of the pilus locus is a clonal property among pneumococcal invasive isolates
Source: BMC Microbiol. 2008 Feb 28;8:41. doi: 10.1186/1471-2180-8-41 (PMC2270847; doi:10.1186/1471-2180-8-41)
Supplement: Additional file 1 — Southern hybridization of a representative set of isolates with the rlrA gene probe. m, lambda PFGE ladder marker (New England Biolabs, Beverly, MA). Lane 1, strain R6. Lanes 2–4, 6 and 8, isolates lacking the pilus islet. Lanes 5 and 9–14, isolates positive for the presence of the rlrA gene. Lane 7, isolate with a weak hybridization signal where the presence of the islet was confirmed by PCR. In all isolates with a negative hybridization result the absence of the pilus islet was confirmed by PCR (see text). [file 1471-2180-8-41-S1.pdf]

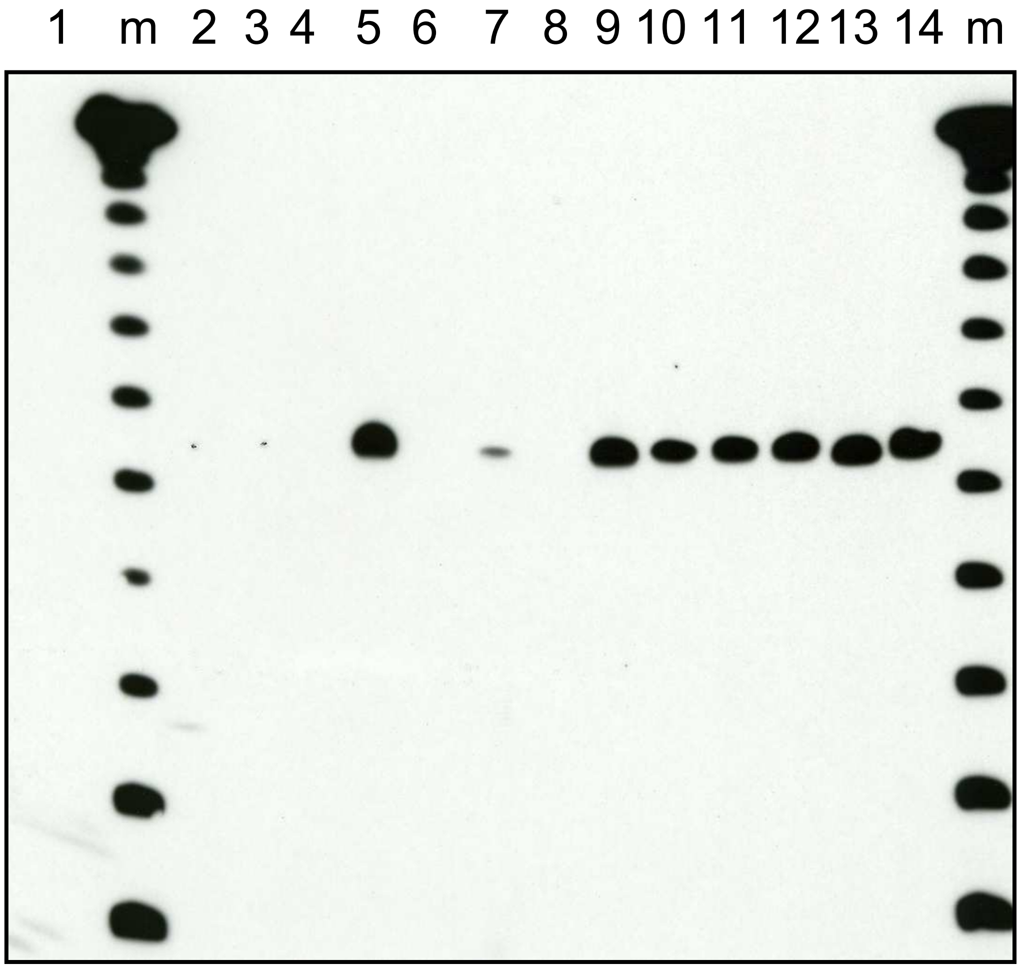

Figure S1. Southern hybridization of a representative set of isolates with the *rlrA* gene probe. m, lambda PFGE ladder marker (New England Biolabs, Beverly, MA). Lane 1, strain R6. Lanes 2-4, 6 and 8, isolates lacking the pilus islet. Lanes 5 and 9-14, isolates positive for the presence of the *rlrA* gene. Lane 7, isolate with a weak hybridization signal where the presence of the islet was confirmed by PCR. In all isolates with a negative hybridization result the absence of the pilus islet was confirmed by PCR (see text).
