# Supplementary material for: The presence of the pilus locus is a clonal property among pneumococcal invasive isolates
Source: BMC Microbiol. 2008 Feb 28;8:41. doi: 10.1186/1471-2180-8-41 (PMC2270847; doi:10.1186/1471-2180-8-41)
Supplement: Additional file 2 — Characteristics of the clones where the pilus locus was identified. [file 1471-2180-8-41-S2.pdf]

Table S1. Characteristics of the serotypes where the pilus locus was identified

Serotype	no. of isolates in serotype and PFGE cluster		STs in PFGE cluster[a]	% rlrA positive isolates (no. isolates)	% of type of sequence among *rlrA* negative strains (no. of isolates)[b]			% of isolates in PFGE cluster resistant to the antimicrobial (no. isolates)			
								Penicillin[c]		Erythromycin	
					A	B	C	All strains	*rlr* A positive strains	All strains	*rlr* A positive strains
**1**	51	33	[ST228 + ST306]	3 (1)	88 (29)	0	9 (3)	0	0	0	0
		17	[ST304 + ST350]	18 (3)	0	0	82 (14)	0	0	6 (1)	0
		1	ND[d]		100 (1)	0	0	0	0	0	0
**3**	46	42	[ST1220 + ST260] + [ST1230 + ST180]	2 (1)	43 (18)	0	55 (23)	0	0	2 (1)	0
		3	ST458	100 (3)	0	0	0	0	0	0	0
		1	ND[d]	100 (1)	0	0	0	0	0	0	0
**4**	29	11	ST247	91 (10)	9 (1)	0	0	0	0	0	0
		13	ST1221	100 (13)	0	0	0	0	0	0	0
		2	ST1222	0	0	0	100 (2)	0	0	0	0
		3	ST1222	0	0	0	100 (3)	0	0	0	0
**6A**	13	2	ST1369	50 (1)	50 (1)	0	0	50 (1)	100 (1)	50 (1)	100 (1)
		3	ST395	0	100 (3)	0	0	0	0	0	0
		2	ST460	0	100 (2)	0	0	0	0	0	0
		6	Other[e]	0	83 (5)	0	17 (1)	0	0	0	0
**6B**	14	8	ST1224 + ST273	100 (8)	0	0	0	12 (1)	12 (1)	88 (7)	88 (7)
		1	ST273	100 (1)	0	0	0	0	0	100 (1)	100 (1)
		2	ST887	0	100 (2)	0	0	100 (2)	0	100 (2)	0
		3	Other[e]	33 (1)	67 (2)	0	0	33 (1)	0	33 (1)	0
**9V**	22	19	[ST156 + ST557+ ST644 + ST1225]	84 (16)	0	16 (3)	0	68 (13)	62.5 (10)	10.52 (2)	12.5 (2)
		2	ST838	100 (2)	0	0	0	100 (2)	100 (2)	0	0
		1	ND[d]	0	100 (1)	0	0	0	0	0	0
**13**	3	1	ST574	100 (1)	0	0	0	100 (1)	100 (1)	0	0
		2	Other[e]	0	100 (2)	0	0	0	0	0	0
**14**	65	2	ST156 +ST1370	50 (1)	50 (1)	0	0	100 (2)	100 (1)	0	0
		49	[ST156 + ST557 + ST790]	98 (48)	0	2 (1)	0	100 (49)	100 (48)	18 (9)	18 (9)
		2	ST156	100 (2)	0	0	0	100 (2)	100 (2)	0	0
		10	[ST15 + ST409]	0	100 (10)	0	0	0	0	60 (6)	0
		2	ND[d]	0	100 (2)	0	0	0	0	0	0
**19A**	17	3	ST416	33 (1)	67 (2)	0	0	0	0	66 (2)	0
		6	ST1201 + ST81	17 (1)	83 (5)	0	0	50 (3)	0	0	0
		2	[ST199 + ST876]	0	100 (2)	0	0	100 (2)	0	0	0
		2	ST276	0	100 (2)	0	0	100 (2)	0	100 (2)	0
		4	Other[e]	75 (3)	25 (1)	0	0	0	0	25 (1)	33 (1)
**19F**	11	4	ST391	25 (1)	25 (1)	0	50 (2)	75 (3)	100 (1)	25 (1)	100 (1)
		1	ST347	100 (1)	0	0	0	100 (1)	100 (1)	0	0
		4	ST177	50 (2)	25 (1)	0	25 (1)	50 (2)	50 (1)	25 (1)	50 (1)
		2	Other[e]	50 (1)	0	0	50 (1)	0	0	0	0
**35B**	1	1	ND[c]	100 (1)	0	0	0	0	0	0	0
**NT**	15	4	ST53	0	100 (4)	0	0	0	0	0	0
		11	Other[e]	36 (4)	36 (4)	0	27 (3)	1	0	0	0
**Total**	**287**			**45 (128)**	**35 (102)**	**1 (4)**	**18 (53)**	**31 (88)**	**55 (69)**	**13 (38)**	**18 (23)**

[a]STs in PFGE clusters with a Dice similarity coefficient of >80%. Brackets indicate STs that belong to the same lineage, as by eBURST analysis with the complete *S. pneumoniae* database available at spneumoniae.mlst.net.

[b]Refer to figure 2 for a discussion of the various types of genetic arrangements found in isolates lacking the *rlrA* islet

[c]Both penicillin intermediate and fully resistant isolates were considered resistant for this analysis.

[d]ND - not determined

[e]Includes several isolates grouped in different PFGE clusters with no MLST information available.
