# Supplementary material for: The presence of the pilus locus is a clonal property among pneumococcal invasive isolates
Source: BMC Microbiol. 2008 Feb 28;8:41. doi: 10.1186/1471-2180-8-41 (PMC2270847; doi:10.1186/1471-2180-8-41)
Supplement: Additional file 3 — Characteristics of the bacterial clones not associated with the pilus locus. [file 1471-2180-8-41-S3.pdf]

Table S2. Characteristics of the serotypes not associated with the pilus locus.

Serotype	no. of isolates in serotype and PFGE cluster		STs in PFGE cluster[a]	% of type of sequence among rlrA negative strains (no. of isolates)[b]			% of isolates in PFGE cluster resistant to the antimicrobial (no. isolates)	
				A	B	C	Penicillin[c]	Erythromycin
**5**	13	12	ST1223	100 (12)	0	0	0	0
		1	ND[d]	100 (1)	0	0	0	0
**7C**	1	1	ND[d]	100 (1)	0	0	0	0
**7F**	18	16	ST191	100 (16)	0	0	0	6 (1)
		1	ST191	100 (1)	0	0	0	0
		1	ND[d]	100 (1)	0	0	0	0
**8**	28	19	ST53	100 (19)	0	0	0	0
		4	ST404	100 (4)	0	0	0	0
		5	Other[e]	100 (5)	0	0	0	0
**9L**	1	1	ND[d]	100 (1)	0	0	0	0
**9N**	10	10	ST66	100 (10)	0	0	0	0
**10A**	9	7	[ST97 + ST1226 + ST1231]	100 (7)	0	0	0	28 (2)
		2	ND[d]	100 (2)	0	0	0	0
**10F**	3	3	ST1649	100 (3)	0	0	0	0
**11A**	7	6	[ST62 + ST408]	100 (6)	0	0	17 (1)	0
		1	ND[d]	100 (1)	0	0	0	0
**11B**	1	1	ND[d]	100 (1)	0	0	0	0
**12B**	16	8	ST218 + ST989	100 (8)	0	0	0	0
		8	[ST218 + ST1365]	100 (8)	0	0	0	0
**15B**	2	2	ST1706	100 (2)	0	0	50 (1)	0
**16F**	4	2	ST414	100 (2)	0	0	0	0
		2	Other[e]	100 (2)	0	0	0	0
**17F**	2	2	ND[c]	100 (2)	0	0	0	0
**18A**	5	3	ST1232		0	100 (3)	0	33 (1)
		2	Other[e]	100 (2)	0	0	0	0
**18C**	12	3	ST1233	100 (3)	0	0	0	0
		1	ST1367	100 (1)	0	0	0	0
		2	ST102	0	0	100 (2)	0	0
		5	ST133 + ST697	100 (5)	0	0	0	0
		1	ND[d]	100 (1)	0	0	0	0
**20**	5	3	ST1026	100 (3)	0	0	0	0
		2	ST235	100 (2)	0	0	0	0
**22F**	12	9	ST1012	22 (2)	0	78 (7)	0	0
		1	ST1372	100 (1)	0	0	0	0
		2	Other[e]	100 (1)	0	0	0	0
**23A**	1	1	ND[d]	100 (1)	0	0	0	0
**23B**	2	2	ND[d]	100 (2)	0	0	0	0
**23F**	24	19	ST338 + ST1371	100 (19)	0	0	89 (17)	0
		3	ST81	100 (3)	0	0	100 (3)	33 (1)
		2	Other[e]	100 (2)	0	0	0	0
**24F**	2	2	ST230	100 (2)	0	0	50 (1)	50 (1)
**25A**	1	1	ND[d]	100 (1)	0	0	0	0
**25F**	1	1	ND[d]	100 (1)	0	0	0	0
**31**	3	3	Other[e]	100 (3)	0	0	0	0
**33A**	1	1	ND[d]	0	0	100 (1)	0	0
**33F**	7	2	ST1367	0	0	100 (2)	0	0
		5	ST717 + ST100	100 (5)	0	0	0	60 (3)
**35F**	3	3	ST1368	100 (3)	0	0	33 (1)	
**36**	1	1	ND[d]	100 (1)	0	0	100 (1)	100 (1)
**38**	1	1	ND[d]	100 (1)	0	0	0	0
**Total**	**196**			**92 (181)**		**8 (15)**	**13 (25)**	**5 (10)**

[a]STs in PFGE clusters with a Dice similarity coefficient of >80%. Brackets indicate STs that belong to the same lineage, as defined by eBURST analysis with the complete *S. pneumoniae* database available at spneumoniae.mlst.net.

[b]Refer to figure 2 for a discussion of the various types of genetic arrangements found in isolates lacking the *rlrA* islet

[b]Both penicillin intermediate and fully resistant isolates were considered resistant for this analysis.

[c]ND - not determined

[d]Includes several isolates grouped in different PFGE clusters with no MLST information available.
